# The New York Homœopathic Union

**Published:** 1891-09

**Authors:** L. M. Stanton

**Affiliations:** Secretary; 71 West 88th St., New York


### THE NEW YORK HOMŒOPATHIC UNION.

### MINUTES OF MEETING OF JUNE 18th, 1891.

The last meeting was held May 21st at Dr. Carleton's office.

It being the Society's third anniversary, Dr. Fincke read an address, which will be found complete in the July number of THE HOMŒOPATHIC PHYSICIAN. He said, speaking of the Koch treatment of tuberculosis, our homœopaths were not carried away or deceived by this new procedure, as the claims of so-called isopathy had already been settled. The homœopaths on the other side of the Atlantic who would have nothing to do with isopathy were now ready to accept it, in order that they might declare Koch's method a homœopathic measure. With us the downfall of this treatment was predicted, and little allusion was made to the extravagance when at its height.

If the nosodes are to be used, they must be obtained, as far as possible, in their purity, potentized and proved upon the healthy, and then they may be considered homœopathic remedies.

But we cannot expect even this fiasco of the Koch lymph to open the eyes of the people when such darkness in medicine prevails among intelligent men and women.

Hahnemann's doctrine should not be judged before perfected, and it was only perfected in the fifth edition of the *Organon* thirty years after the first.

All reformers were at first reviled, and such men as Copernicus, Kepler, and Newton had to encounter the enmity of their contemporaries.

There is this difference between homœopathic and old-school medicine. The so-called progress of the old school means progress in the auxiliary sciences of medicine, but because there are continually accumulating facts and knowledge, it does not necessarily follow that there is progress in medicine. New measures are introduced in medicine which should remain where they belong, as they turn out to be useless in the treatment of the sick. The science of Homœopathy proceeds on its way pursuing the perfection of its own methods, regardless of the ever-changing hypotheses and theories of the physico-chemical school.

Following Dr. Fincke's address, sections 103–109 of the *Organon* were read.

Were all Hahnemann's provings with the low potencies and crude drugs? was asked. Hahnemann proved Carbo-veg., Lycopodium, and Nat-mur. high, Carbo-veg. in the 30th potency.

In section 108, Dr. Fincke's translation, the words, "in moderate quantities," should be inserted after "single medicines."

The old school was taken to task for its presumption in styling itself scientific. It certainly cannot be called scientific because its practice is based upon the sciences of physics and chemistry. It might be made up of a dozen different sciences, yet if it lacks the spirit of science, which is the investigation of truth for truth's sake, it cannot be called scientific.

What is the reason that some men who have used the high potencies have abandoned them and condemned them? In answer to this question it was remarked that we all lose faith in what we do not continually practice, hence men who use mostly the low potencies, and only occasionally the high, have little faith in the high potencies, and as loss of faith is practically equivalent to condemnation, the high potencies are condemned because not used and tested.

L. M. STANTON,

*Secretary.*

71 WEST 88th ST., NEW YORK.

